# Nonlinear optical imaging by detection with optical parametric amplification (invited paper)

**DOI:** 10.1142/s1793545822450018

**Published:** 2022-11-21

**Authors:** Yi Sun, Haohua Tu, Stephen A. Boppart

**Affiliations:** * Beckman Institute for Advanced Science and Technology, University of Illinois at Urbana-Champaign, Urbana, IL 61801, USA; † Department of Electrical and Computer Engineering, University of Illinois at Urbana-Champaign, Urbana, IL 61801, USA; ‡ Carle Illinois College of Medicine, University of Illinois at Urbana-Champaign, Urbana, IL 61801, USA

**Keywords:** Nonlinear optical microscopy, optical parametric amplification, optical detection

## Abstract

Nonlinear optical imaging is a versatile tool that has been proven to be exceptionally useful in various research fields. However, due to the use of photomultiplier tubes (PMTs), the wide application of nonlinear optical imaging is limited by the incapability of imaging under ambient light. In this paper, we propose and demonstrate a new optical imaging detection method based on optical parametric amplification (OPA). As a nonlinear optical process, OPA intrinsically rejects ambient light photons by coherence gating. Periodical poled lithium niobate (PPLN) crystals are used in this study as the media for OPA. Compared to bulk nonlinear optical crystals, PPLN crystals support the generation of OPA signal with lower pump power. Therefore, this characteristic of PPLN crystals is particularly beneficial when using high-repetition-rate lasers, which facilitate high-speed optical signal detection, such as in spectroscopy and imaging. A PPLN-based OPA system was built to amplify the emitted imaging signal from second harmonic generation (SHG) and coherent anti-Stokes Raman scattering (CARS) microscopy imaging, and the amplified optical signal was strong enough to be detected by a biased photodiode under ordinary room light conditions. With OPA detection, ambient-light-on SHG and CARS imaging becomes possible, and achieves a similar result as PMT detection under strictly dark environments. These results demonstrate that OPA can be used as a substitute for PMTs in nonlinear optical imaging to adapt it to various applications with complex lighting conditions.

## 1. Introduction

Nonlinear optical imaging technologies play a key role in generating many of the significant findings of biological research.^1–6^ In a nonlinear optical imaging system, the intense optical power achieved by ultrafast lasers and high-numerical-aperture objective lenses stimulate various nonlinear optical processes within biological samples.^2^ Other than the high optical power, nonlinear optical processes also have a selective dependence on the sample characteristics, including sample structure and chemical components.^7–10^ The selective dependence, in turn, provides different forms of contrast for nonlinear optical imaging. With these forms of contrast, nonlinear optical imaging has become particularly suitable for the investigation of *ex vivo* and *in vivo* biomedical samples free of any labeling or contrast agents.^4,11,12^ However, compared to the optical signals generated from labeling agents, label-free nonlinear optical signals from biomedical samples are orders-of-magnitude weaker, ruling out the possibilities of using camera sensors or photodiodes as the detection method.

The detection of nonlinear optical imaging predominantly relies on the use of photomultiplier tubes (PMTs).^4–6^ A PMT is a highly sensitive optical detector that multiplies the number of photoelectrons based on secondary emission effects. Most commercial PMTs can achieve a gain ratio ranging from 10^5^ to 10^6^, which has proven to be sufficient to bring a label-free nonlinear optical signal up to a detectable level by the electronics.^13^ In high-speed laser scanning microscopy, PMTs provide much higher sensitivity and detection speed over other sensitive optical detectors like avalanche photodiode and Electron-Multiplying CCDs (EMCCDs), and therefore are predominantly used in nonlinear optical imaging applications.

However, PMTs also have shortcomings. First, the use of photosensitive semiconductor material remarkably compromises the sensitivity of the PMT in the near-infrared spectral region, where each photon carries less energy.^14,15^ The sensitivity of a PMT decreases by two orders-of-magnitude by changing the detection wavelength from 400 nm to 1000 nm.^13^ Another major shortcoming of PMTs is the incapability to reject ambient light. Based on the nature of PMT detection, a photon originating from a room light bulb or a small LED indicator has the same chance of knocking out an electron from a PMT cathode as a signal photon from an imaging system. Our previous work demonstrated the possibilities of fully concealing the imaging system.^6^ To avoid the effects of ambient light, even the venting grill is fully covered. However, it was found that long-term imaging acquisition would inevitably cause over-heating of the system.^6^ In addition, the concealment of the system makes it impossible to manipulate the sample while monitoring, such as adding chemicals and finding surgical marks. Due to these shortcomings, PMTs and PMT-based nonlinear optical imaging systems are primarily operated in strictly dark environments for optical detection within the ultraviolet to visible spectral range.

To overcome these limitations for nonlinear optical microscopy, previous works demonstrated various methods to reject the ambient light while preserving the majority of the imaging signal in nonlinear optical microscopy.^16–18^ First, the most straightforward approach is by spectrally filtering the narrowband nonlinear optical imaging signals like those from second harmonic generation (SHG) and coherent anti-Stokes Raman scattering (CARS).^18^ However, spectral filtering is insufficient to attenuate the ambient light without sacrificing desired imaging signal intensity. For instance, in femtosecond SHG imaging, a 10 nm bandpass spectral filter only provides about 15 dB optical attenuation to the 300 nm-wide visible spectra of most white LEDs lights. In our previous studies of intraoperative nonlinear optical imaging, a 20 nm narrow bandpass spectral filer was placed tightly in front of a PMT for SHG signal detection.^5,6^ Without other concealment, the residual ambient light still easily saturated the PMT. This disproves the idea of using spectral filtering alone for ambient light rejection in nonlinear optical imaging.

The second approach is by digitally modulating the imaging signal and using a lock-in amplifier to extract the modulated signal.^18^ This method works efficiently if the ambient lighting environment is single-sourced and well controlled. However, in most realistic lighting environments like the hospital, multiple light sources with different frequencies and delays typically exist. In addition, the use of digital modulation and lock-in amplification is highly sensitive to the stability and noise of the pulsed pump laser. Finally, the work^18^ by Zhang *et al*. combined the use of spectral filtering and digital modulation, but only produced one label-free CARS image of a biological sample with no comparison with the PMT-detected image within a dark environment. Therefore, the digital modulation solely, or combined with spectral filtering, still cannot completely overcome the limitations of the PMTs in nonlinear optical imaging.

Another method of ambient light rejection is heterodyne detection. As employed in optical coherence tomography (OCT), heterodyne detection can reach the shot-noise limit.^19–22^ In addition to OCT, heterodyne detection has also been used in nonlinear optical imaging, such as SHG and CARS, to unravel the phase information of these nonlinear optical imaging signals. However, there are very few works dedicated to use interferometry for ultra-sensitive nonlinear optical imaging detection and to replace the use of highly sensitive optical detectors like the PMTs. M. Jurna *et al*. presented their feasibility study of using heterodyne in CARS signal detection.^17^ Digital modulation and specific detection bandwidth were deliberately designed in their work to achieve a shot-noise-limited detection performance. However, no following work has been reported in any biomedical CARS imaging applications to further prove the concept of this study. In addition, as the imaging wavelength of CARS and other nonlinear optical imaging modalities ranges from ultraviolet to near infrared, it is difficult for heterodyne detection to generate different reference signals to accommodate such a wide range of detection wavelengths.

In this paper, we propose the use of an alternative optical amplification and detection method for nonlinear optical imaging in place of PMTs, which is based on optical parametric amplification (OPA). OPA, as a nonlinear optical imaging process, describes the splitting of a pump photon into a signal photon and an idler photon, which is stimulated by an input photon at the signal wavelength.^23,24^ With a strong pump, the OPA process can amplify an extremely weak seed down to the quantum noise level by a large gain ratio.^25–29^ In particular, due to the nature of OPA and nonlinear optical processes in general, the involving signals are required to lie within a certain range of spatial direction, temporal delay, polarization, wavelength, etc. While the weak yet highly coherent imaging signals easily satisfy these requirements, only an extremely small portion of the ambient light photons meet all the requirements above. Therefore, the absolute majority of the ambient light photons are rejected by the gating mechanisms of OPA. For instance, the temporal gating provided by femtosecond laser pulses can effectively attenuate the ambient light by at least 60 dB, which means only one in a million ambient light photons can pass the temporal gate.

In addition to this ambient-light-rejecting capability, previous works have shown the sensitivity of OPA in ultraweak signal detection and amplification to approach the shot-noise limit.^25–27,30^ However, limited by the conversion efficiency of the barium borate (BBO) crystal as the OPA medium, they exclusively use low-repetition-rate (~10 Hz) lasers to boost the peak power of the pump.^25,27,28^ For nonlinear optical imaging, low repetition rates inevitably result in an unacceptably slow imaging speed that fails to support realistic imaging applications. Our recent work explored the possibility of using OPA for high-speed detection of linear reflectance confocal imaging.^15^ The OPA employed in that work was based on a periodical poled lithium niobate (PPLN) crystal which offers a great advantage over a BBO crystal, requiring much lower pump power. At a megahertz repetition rate, the OPA detection outperformed a commercial near-infrared (NIR) PMT in the NIR spectral range, which overcomes the detection sensitivity limitation of the PMT in the NIR. Compared to the linear reflectance imaging, nonlinear optical imaging provides more information on the microscopic structure and molecular components of the biomedical sample, and the nonlinear optical imaging signal is generally orders-of-magnitude weaker. Therefore, there is certainly higher impact and great necessity in using OPA detection for nonlinear optical imaging.

In this paper, the concept of high-speed OPA detection is demonstrated for two nonlinear coherent optical imaging modalities, SHG and CARS imaging. Since the emission signals of these two imaging modalities are highly coherent with respect to the imaging excitation source, they can be detected by OPA with higher efficiencies. PPLN crystals are used in this study to generate the excitation wavelengths for SHG and CARS imaging, and more importantly, serve as the OPA media for the detection of the emission signal from SHG and CARS imaging. The laser parameters are optimized for both imaging excitation and OPA detection. Using OPA detection, we demonstrated ambient-light-on SHG and CARS microscopy imaging with comparable signal-to-noise ratio and imaging depth performance as the PMT-detected images under strictly controlled dark conditions. The imaging results strongly suggest that OPA detection can be employed in place of PMTs to perform nonlinear optical imaging under complex lighting conditions. We believe that OPA-detected nonlinear optical imaging will advance the future clinical translation of nonlinear optical imaging technologies.

## 2. Materials and Methods

**Fig. 1.**
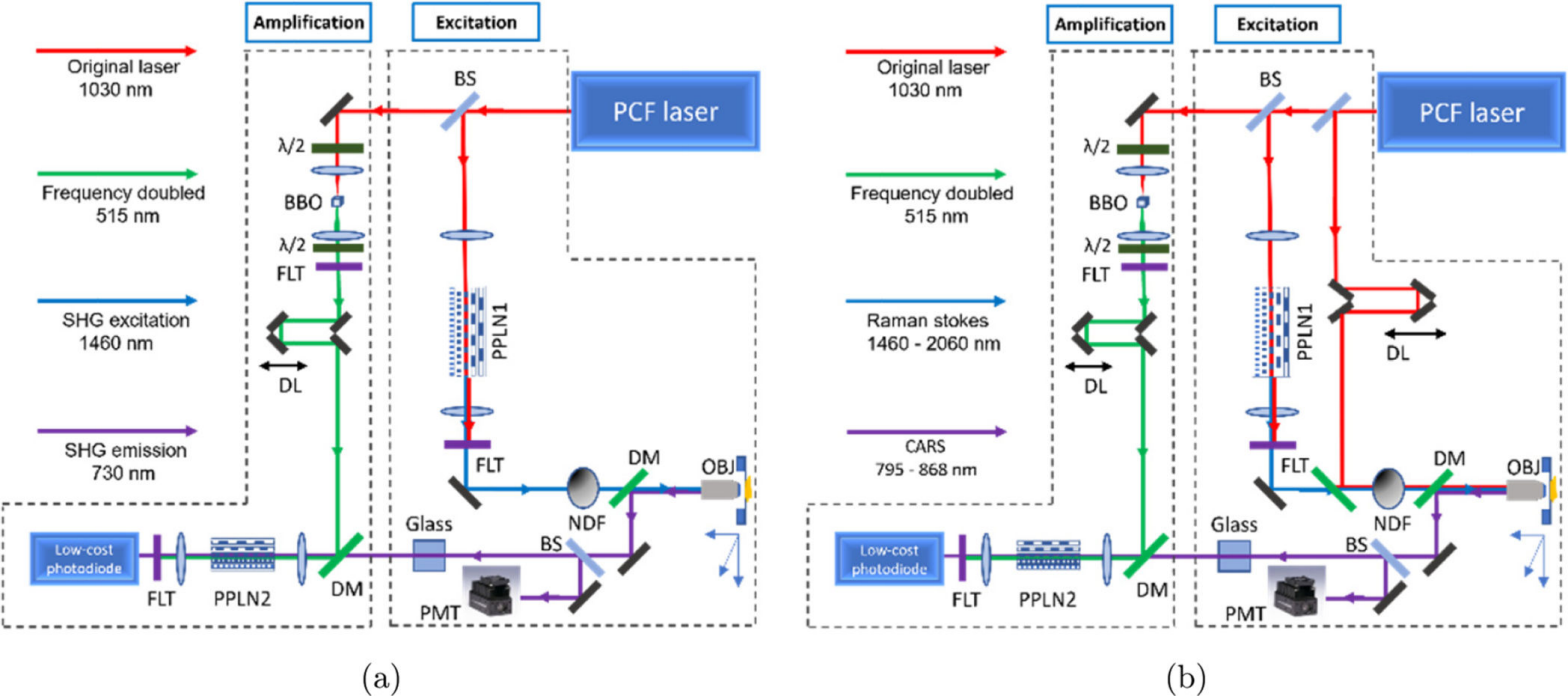
Schematics of the OPA-detected nonlinear imaging systems. (a) SHG-OPA microscope. (b) CARS-OPA microscope. The wavelengths in each system are represented by different line colors, and indicated in the legends on the left side of each. λ/2: half-wave plate. BS, beam splitter; DL, delay line; DM, dichroic mirror; FLT: optical filter; NDF, neutral density filter; OBJ, objective; PM, prism. PCF: photonics crystal fiber.

The system setup for OPA-detected SHG and CARS imaging is shown in Figs. 1(a) and 1(b), which can both be summarized as the separate creation of the OPA pump and imaging excitation along with the combination of the OPA pump and the imaging emission. To begin with, the system schematic of OPA-detected SHG imaging shown in Fig. 1(a) is divided into two parts by dashed regions. The right region of the system contains the optics for excitation generation and imaging, while the left region includes the OPA detection elements of the system.

In the SHG-OPA system in Fig. 1(a), a photonic crystal fiber (PCF) tunable laser (Satsuma, Amplitude Laser Inc.) was used as the laser source for both the imaging and the OPA elements. The flexibility offered by the tunable PCF laser includes an adjustable repetition rate from 500 kHz to 10 MHz and tunable chirping to expand the pulse duration from 340 fs to about 2 ps. In the excitation part of the system, the PPLN1 (MOPO1–1.0–10, Covesion, Ltd.) took the focused 1030 nm laser output as the pump and generated the excitation signal for SHG microscopy imaging via the optical parametric generation (OPG) process. Through spectral anti-reflection coating and temperature tuning, the OPA signal output of PPLN1 ranges from 1460 nm to 2060 nm if using a 1030 nm pump. In the amplification part of the system, another type of PPLN, named PPLN2 in Fig. 1(a), is used to amplify the imaging emission signal. The major difference between PPLN1 and PPLN2 is the length of their poling periods. Designed to amplify visible and near-infrared wavelengths, the PPLN2 (MOPO515–0.5–10, Covesion, Inc.) has shorter poling periods (6–8.36 *μ*m) than those of the PPLN1 (29.52–31.59 *μ*m). In addition, the anti-reflection coating of PPLN2 is also optimized for visible to near-infrared wavelengths. The designed pump wavelength for the PPLN2 is 515 nm, and corresponding OPA signal output ranges from 640 to 1030 nm.

In this study, the excitation and emission wavelengths of SHG imaging must lie within the OPA signal output range of PPLN1 and PPLN2, respectively. In addition, since the SHG response of the collagen fibers to the excitation wavelengths continuously decreases after approximately 890 nm,^31^ we chose 1460 nm, the shortest PPLN1 output wavelength, as our SHG excitation. Accordingly, the SHG emission wavelength of 730 nm lies within the amplifiable range of PPLN2.

The generated SHG excitation from PPLN1 was collimated by a lens and attenuated by an adjustable neutral density filter (NDF) before being directed into a stage-scanning microscope. After reaching the sample, the backward SHG emission at 730 nm was collected and collimated by the objective lens. The SHG emission signal was separated from the path of the excitation by a dichroic mirror (DM). A 50/50 beam splitter divided half of the SHG signal photons and sent them to a PMT (PMT1001, Thorlabs, Inc) as a reference to compare with the OPA detection. The emission beam passed through several SF57 glass cubes with different lengths (2.5, 5, and 10 inches), so the pulse duration of the emission signal could be independently adjusted by changing the total lengths of the glass cubes. With a transform-limited pulse duration of 340 fs, by alternating the length of the glass cubes, the pulse duration of the emission signal can be roughly adjusted from 340 fs to about 850 fs.

The OPA part of the OPA-detected SHG imaging system consisted of a frequency doubling BBO crystal, an optical delay line, and OPA optical elements. To generate the 515 nm OPA pump, the 1030 nm laser output was focused into a BBO crystal for frequency doubling. In Fig. 1(a), the half-wave plate before the BBO crystal was used to achieve maximal SHG conversion efficiency, while the one after the BBO crystal aims to realign the polarization of the 515 nm output with the OPA input signal. An optical delay line was added after the frequency-doubling setup to synchronize the OPA pump with the SHG emission signal from the imaging portion of the system. Following this, the synchronized OPA pump (515 nm) and signal (730 nm) were spatially combined using a dichroic mirror and focused into PPLN2. Finally, the SHG imaging signal was amplified by PPLN2 and detected by a biased photodiode (DET10A, Thorlabs, Inc.).

The photocurrent output of the photodiode was digitized and processed by a PCIe digitizer board (ATS9360–4G, Alazar Technologies Inc.) on a PC. To assure accurate pixel registration, the digitizing and processing of the photodiode signal was synchronized with the scanning stage by sending the trigger signal from the stage controller to the digitizer board via a BNC cable. For each scanning line, the digitizer was triggered by the TTL signal sent from the stage controller to start data acquisition. Upon the completion of data acquisition for each scanning line, the signal intensities at the photocurrent pulse peaks were extracted from the raw data by intensity thresholding, and the extracted peak values within the dwelling time of each pixel were averaged to represent the pixel value. Limited by the stage scanning speed and stability, we used a pixel dwelling time of 5 ms and a pixel size of 1 *μ*m. To assure a fair comparison with the PMT, the PMT acquisition followed the same signal processing strategy.

The elements for the OPA-detected CARS imaging system shown in Fig. 1(b) are only different from the OPA-detected SHG imaging system by the presence of an extra beam path added in the excitation part of the system, which is used for the CARS pump. In Fig. 1(b), the pump beam for CARS is obtained directly from the laser output, while the Stokes beam is generated from the OPG process in PPLN1, as for the SHG excitation. The tunable output of PPLN1 offers a range of Raman wavenumbers from 2878 cm^−1^ to 4854 cm^−1^.

## 3. Results

### 3.1. Ambient-light-on SHG imaging by OPA

An SHG microscope was built to demonstrate OPA detection of the coherent SHG imaging signal. With a tunable repetition rate and pulse duration, the optimal imaging parameters used for SHG imaging and OPA detection were found to be a 500 kHz repetition rate, a 320 fs OPA pump pulse duration, a 400 fs imaging excitation pulse duration, and a 650 mW OPA pump. These parameters were empirically determined by optimizing the performance of OPA and SHG imaging. The optical power of the imaging excitation beam varied according to the imaging samples.

To characterize the OPA imaging performance, the SHG signal generated from a BBO crystal was detected by OPA and PMT. By attenuating the power of the SHG pump, the OPA and PMT detection both reached a detection limit of approximately 100 pW, or 200 aJ per pulse.

**Fig. 2.**
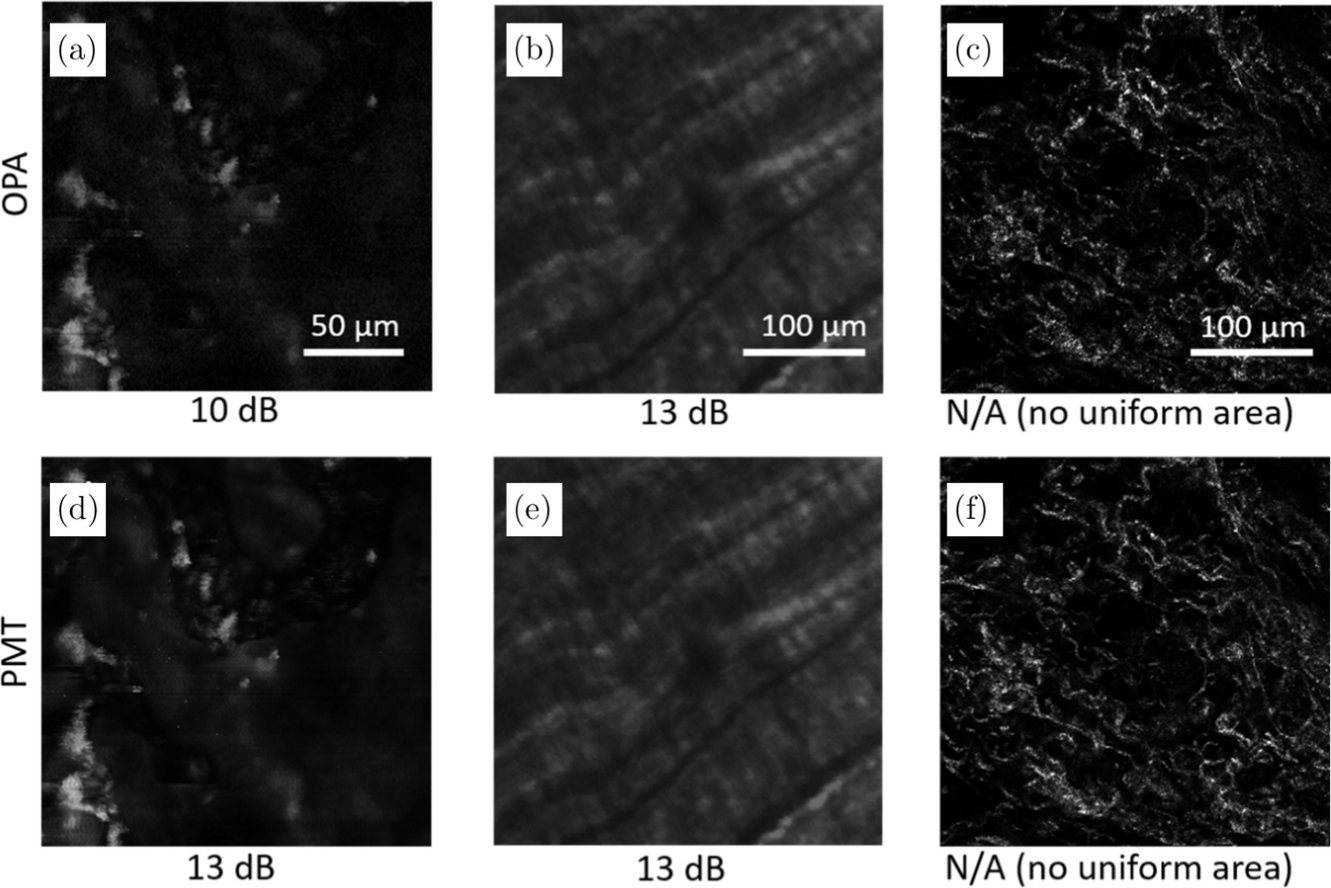
Comparison of OPA- and PMT-detected images taken from the same image site of a ((a), (d)) potato slice, ((b), (e)) chicken tendon, and ((c), (f)) rat mammary tissue. The SNR of each image is listed below the corresponding image.

With the same image excitation power of 40 mW, and using the OPA parameters mentioned earlier in this section, OPA- and PMT-detected SHG images were collected from different biological samples, including a potato slice, chicken tendon, and the collagen fibers from formalin-fixed rat mammary tissue. The comparison between PMT and OPA detection is shown in Fig. 2. To reflect the actual signal strength and SNR, the intensities of the images in Fig. 2 were not normalized. It was found that the SNRs for the OPA-detected images of the potato slice and chicken tendon reached the level of the PMT-detected images. However, it is difficult to calculate the SNR from the images collected from the rat mammary tissue due to the lack of large areas with a relative uniform signal and background. Nonetheless, the fibrous structure of the mammary tissue was visualized with a similar number of details by both image detection methods.

**Fig. 3.**
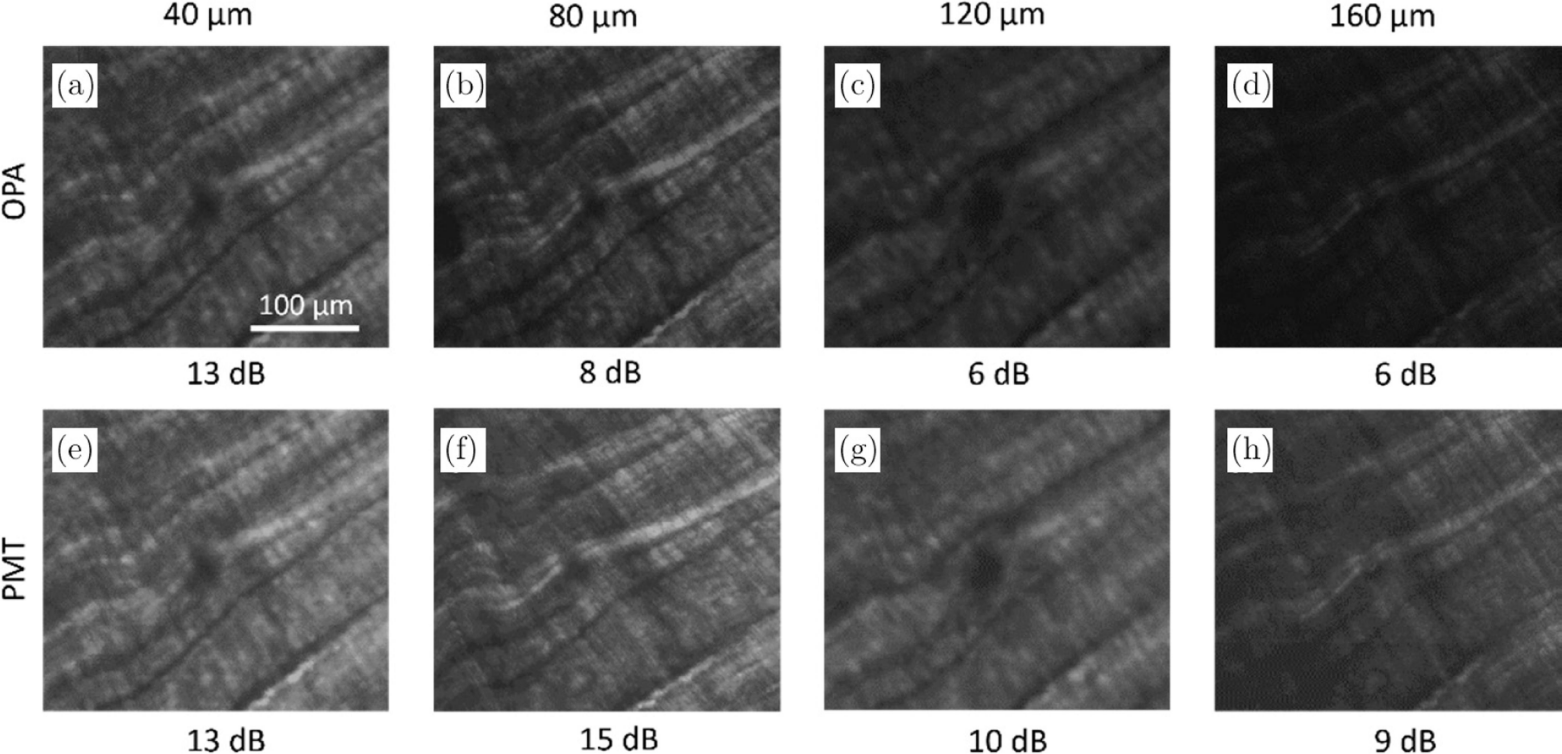
Depth-resolved imaging of chicken tendon tissue using (a)–(d) OPA and (e)–(h) PMT detection.

In addition, the depth-resolved imaging ability of OPA detection was demonstrated. Figure 3 shows depth-resolved images of fresh chicken tendon tissue collected separately when using OPA or a PMT, spanning the imaging depth from 40 *μ*m to 200 *μ*m. To maximize the OPA gain, the delay line of the OPA pump was readjusted every time when the focal position along the *z* axis was changed. As seen in Fig. 3, the images collected using the OPA share similar image quality and visualize similar image features as the images collected using the PMT. It is noticed that the SNR of the OPA-detected images differs more from the PMT-detected images at greater depths. It should be noted that all the OPA-detected SHG images were collected with ambient light present, meaning that a ceiling light of ~1600 Lumens was kept on during image collection. On the other hand, the PMT was used to collect images with all the light sources being turned off or blocked, including the monitor screen, the power indicator on the laser, and the LED indicator on the desktop computer. We found that even the indirect illumination from a 3 mm LED indicator within the room would cause remarkable noise on the PMT signals. Therefore, the PMT provided slightly better image performance if operated in the strictly dark environment, while the OPA has an absolute advantage in ambient-light-on image collection without substantially sacrificing image SNR.

### 3.2. Ambient-light-on CARS imaging by OPA

**Fig. 4.**
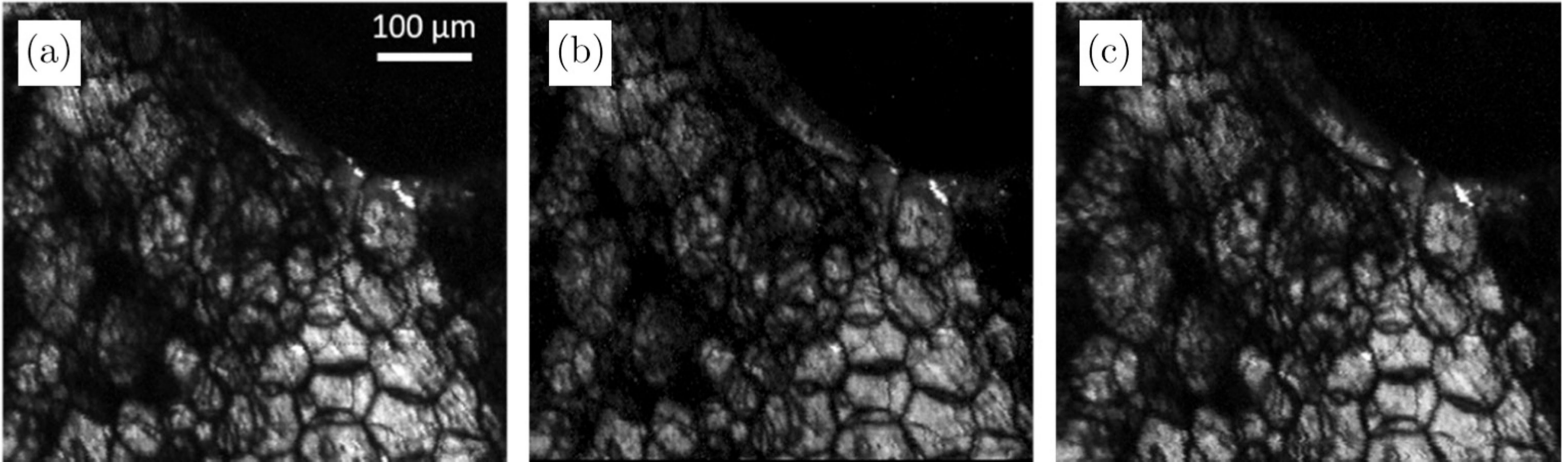
CARS images collected from the same imaging site in pig adipose tissue with (a) PMT detection using an 18 mW pump beam and a 12 mW Stokes beam, (b) OPA detection using a 30 mW pump beam and a 16 mW Stokes beam with no ambient light present, and (c) OPA detection using a 30 mW pump beam and a 16 mW Stokes beam by OPA with ambient light present.

After the demonstration of SHG-OPA imaging, the use of OPA detection was further explored with CARS imaging of biological tissues. Using a 1030 nm pump and a 1460 nm Stokes signal, we aimed to probe a wavenumber of 2859 cm^−1^, which lies within the CH lipid region of adipose tissue. Figure 4 shows images from pig adipose tissue under different conditions. Figure 4(a) is the reference image collected by a PMT with an 18 mW pump beam and a 12 mW Stokes beam. It is obvious that the two images (Figs. 4(b) and 4(c)) collected by OPA with and without ambient light present are highly similar, indicating that the ambient light has negligible effect on the OPA detection. Turning on any ambient light while using PMT detection would result in saturation of the PMT, and potentially catastrophic damage. A slightly higher pump (30 mW) and Stokes power (16 mW) was needed for the OPA detection to achieve a similar image SNR as the PMT detection, which used an 18 mW pump and a 12 mW Stokes power. To be noticed, the slight increase of the input power for OPA detection is much less significant than the total gain ratio of the OPA.

**Fig. 5.**
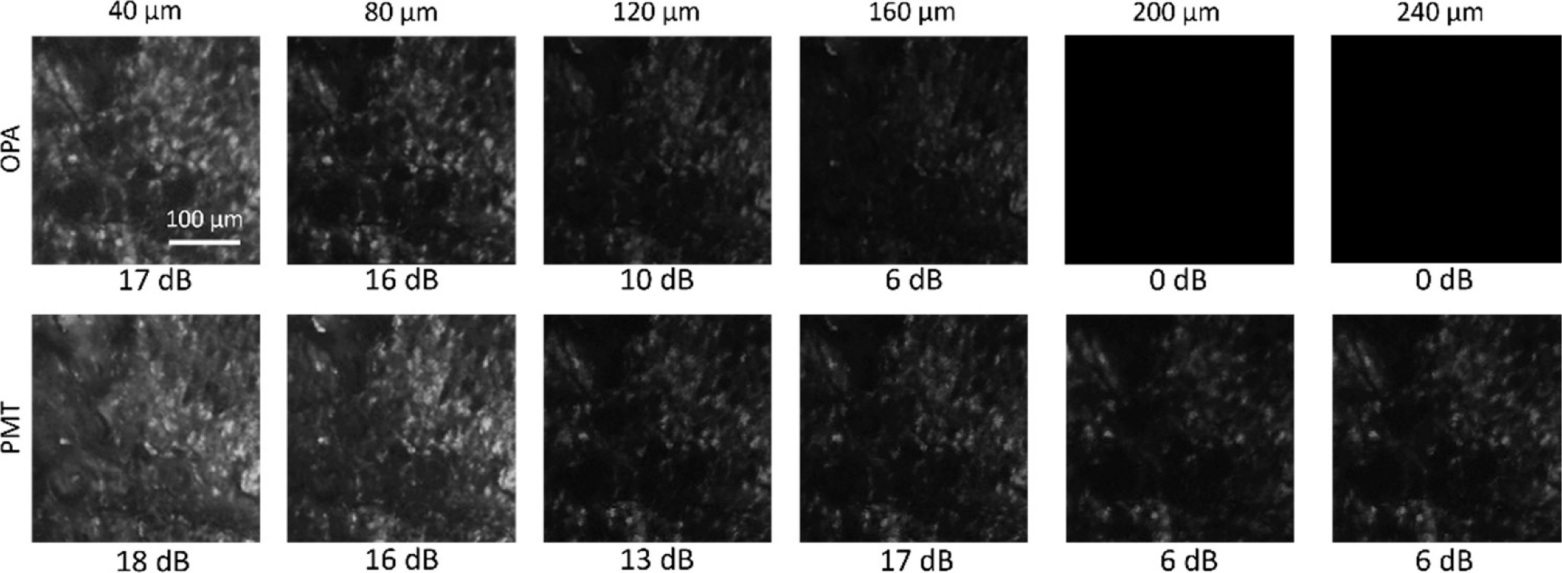
Depth-resolved imaging of pig adipose tissue detected by OPA (upper row) and PMT (lower row). The image SNR is listed below each corresponding image. The OPA-detected CARS images stop showing any tissue features beyond 160 *μ*m.

Figure 5 shows depth-resolved images collected using OPA and PMT detection. The excitation parameters for OPA and PMT detection are the same as in Fig. 4. While the PMT detection managed to visualize tissue features down to a depth of 240 *μ*m, the OPA detection ceased to show any feature contrasts deeper than 160 *μ*m. It is also noticed that the image SNRs for the two detection methods are similar at shallower depths, but the image SNR with OPA detection rapidly dropped with increasing image depth. Similar to the depth-resolved imaging results in OPA-detected SHG, this is partially because the scattered photons from the deeper tissue regions carry a wider range of phases/temporal delays compared to the ballistic imaging photons at a shallower depth, and therefore are not efficiently amplified by the OPA process.

## 4. Discussion

**Fig. 6.**
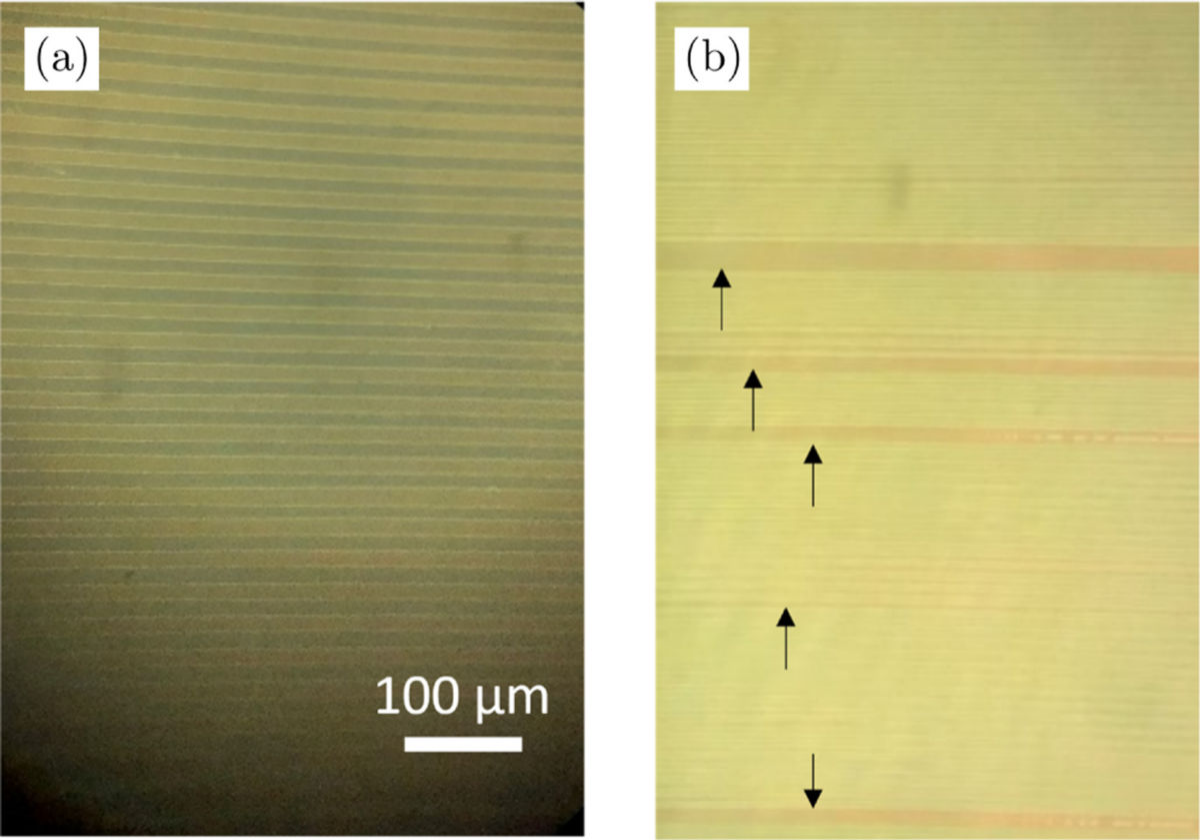
The microscopy image of (a) a 31 *μ*m and (b) a 6 *μ*m PPLN channel. The scale bar is applied to both microscopy images. The defects in the 6 *μ*m PPLN channel are pointed out by black arrows.

As a pilot study in this new field, there certainly are a few future improvements and unexplored areas related to the results presented in this work. First, the performance of OPA detection for nonlinear optical imaging did not reach the performance of the previously demonstrated OPA detection of NIR linear reflectance confocal imaging.^15^ This can be attributed to the use of different PPLNs. While the PPLN used for linear reflectance confocal imaging was pumped by a 1030 nm laser and had a poling period of around 31 *μ*m, the PPLN crystal used in this study relies on a shorter pump wavelength at 515 nm that requires a shorter poling period of approximately 6 *μ*m. The rather small difference in poling period presents a significant manufacturing challenge that results in a number of defects in the poling structure, as shown in Fig. 6. These defects affect the conversion efficiency of the PPLN-based OPA system. In addition, like most dielectric optical materials, the PPLN has a greater dispersion at shorter wavelengths. Therefore, the pump and signal pulses in this study have a shorter temporal overlap when travelling through the PPLN crystal. We believe these issues with the PPLN crystals can be mitigated in the future with the advancing PPLN manufacturing technology. Second, it is noticed that the performance for OPA-detected CARS is lower than for OPA-detected SHG. This can be partially attributed to the coherence of the emission signal. While the CARS signal is considered coherent, it is still dependent on the vibrational energy levels of the molecules. The beam shape and the temporal distribution of the CARS signal might not be as ideal as the SHG signal, which is generated from a parametric process. Third, the imaging speed of this study is insufficient to capture real-time tissue dynamics. This is mainly limited by the stable scanning speed of the translational stage. Because of the temporal matching between the imaging signal and OPA pump, the axial position of the sample is crucial for our OPA detection. We believe the speed limitation can be improved by faster and more stable galvanometer mirror scanning. Fourth, the optical signal that can be amplified by OPA is narrowband compared to the broadband PMT detection. Therefore, imaging signals that span a wide spectral range cannot be effectively amplified and detected by OPA. However, we want to reiterate that the OPA detection is easily tunable to accommodate different wavelengths by adjusting the temperature and period length of the PPLN crystal.

## 5. Conclusion

To conclude, we demonstrated the capabilities of OPA detection in high-speed nonlinear optical imaging signal detection and the potential for replacing the widely used PMT to enable ambient-light-on nonlinear optical imaging. Recently developed PPLN crystals were employed as OPA media in place of the bulk nonlinear crystals like BBO, and offer a higher conversion efficiency than conventional bulk nonlinear optical crystals at a lower peak power. By optimizing the laser parameters for OPA, SHG and CARS imaging signals were successfully detected by OPA, even with ambient light present, demonstrating an imaging performance comparable to PMT detection in strictly dark environments. In particular, this work distinguished itself from previous works on OPA detection by operating at a 500 kHz repetition rate, which supports high-speed and near-real-time imaging performance. The OPA-detected images were collected from various tissue samples with similar imaging performance, demonstrating the versatility of the OPA detection. We believe these results suggest that OPA detection can be readily used as a detection method for nonlinear optical imaging where weak imaging signals can be detected under ambient light conditions, such as in biological labs or clinical settings.
